# Effects of psoralen on the pharmacokinetics of anastrozole in rats

**DOI:** 10.1080/13880209.2018.1501584

**Published:** 2018-10-21

**Authors:** Yuzhu Zhang, Jingjing Wu, Yue Zhou, Yulian Yin, Hongfeng Chen

**Affiliations:** TCM Department of Breast, Longhua Hospital Affiliated to Shanghai University of TCM, Shanghai, China

**Keywords:** LC-MS/MS, CYP3A4, *P-gp*

## Abstract

**Context:** Psoralen and anastrozole are always used together for breast cancer patients in Chinese clinics.

**Objective:** This study investigates the effects of psoralen on the pharmacokinetics of anastrozole in rats and its potential mechanism.

**Materials and methods:** The pharmacokinetics of orally administered anastrozole (0.5 mg/kg) with (test group) or without (Control group) psoralen pretreatment (20 mg/kg/day for 10 days) in male Sprague-Dawley rats (six rats in each group) were investigated. The plasma concentration of anastrozole was determined using a sensitive and reliable LC-MS/MS method. Additionally, the effects of psoralen on the intestine transport and metabolic stability of anastrozole (1 μM) were investigated using a Caco-2 cell transwell model and rat liver microsome incubation systems.

**Results:** The results indicated that psoralen could significantly increase the *C*_max_ (from 56.74 ± 3.17 ng/mL to 83.26 ± 6.87 ng/mL), and *t*_1/2_ (from 10.80 ± 1.05 to 14.29 ± 1.38 h) of anastrozole (*p* < 0.05). Psoralen could also significantly decrease the efflux ratio of anastrozole from 1.88 to 1.32 (*p* < 0.05). Additionally, the intrinsic clearance rates of anastrozole decreased significantly (from 62.83 to 43.97 μL/min/mg protein) (*p* < 0.05) with psoralen pretreatment in rat liver microsome incubation systems.

**Discussion and conclusions*:*** This study indicates that when the rats were pretreated with psoralen, the system exposure of anastrozole would be increased significantly. The results showed that the herb-drug interaction between psoralen and anastrozole might occur when they were co-administered, and future studies in humans also need to investigate its herb-drug interaction potential.

## Introduction

Anastrozole is a derivative of benzotriazole marketed as ARIMIDEX^®^ by AstraZeneca Pharmaceuticals LP, and it has an inhibitory action on aromatase, thus blocking the conversion of testosterone into estradiol and androstenedione into estrone (Wilkinson 2004; Ding et al. 2017; Ellis 2017; Nave et al. 2018). Anastrozole has significant effects on breast cancer treatment, and it is currently used as first-line treatment in estrogen receptor (ER)-positive postmenopausal women, particularly to treat locally advanced or metastatic breast cancer (Wellington and Faulds 2002; Sanford and Plosker 2008; Kelly and Buzdar 2010). Anastrozole is oxidized to hydroxyanastrozole mainly by CYP3A4 (Antoniou and Tseng 2005; Kamdem et al. 2010; Abubakar et al. 2014), and the transport of anastrozole is mediated by *P-gp*, which limited its absorption at the blood–brain barrier and intestine (Miyajima et al. 2013).

Psoralen is the main active ingredient extracted from the natural products of *Psoralea corylifolia* L. (Leguminosae) and it has been commonly used in traditional Chinese medicine for the treatment of osteoporosis, osteosarcoma, bone fracture and osteomalacia (Hao et al. 2011; Chen et al. 2017; Du et al. 2017; Kassahun Gebremeskel et al. 2017). Liu and Flynn (2015) found that psoralen could inhibit the activity of CYP3A4 in a concentration dependent manner. Jiang et al. (2016) reported that psoralen could inhibit the efflux function mediated by *P-gp*, which may be important for increasing the efficiency of chemotherapy and improving the clinical protocols aiming to reverse *P-gp*-mediated MDR. Therefore, herb-drug interaction mediated by CYP3A4 or *P-gp* should be cautioned when psoralen was co-administered with CYP3A4 or *P-gp* substrate. As we know, psoralen and anastrozole are always used together in breast cancer patients in Chinese clinics, and however, the herb-drug interaction between psoralen and anastrozole is still unknown, especially the effects of psoralen on the pharmacokinetics of anastrozole.

This study investigates the effects of psoralen on the pharmacokinetics of anastrozole in rats. First, the *in vivo* pharmacokinetics of anastrozole in rats with or without psoralen pretreatment were determined using a sensitive and reliable LC-MS/MS method, and then, the effects of psoralen on the transport of anastrozole were investigated in the Caco-2 cell transwell model. Finally, the effects of psoralen on the metabolic stability of anastrozole were determined using rat liver microsome incubation systems.

## Materials and methods

### Chemicals and reagents

Anastrozole (purity >98%), omeprazole (purity >98%) and psoralen (purity >98%) were purchased from Shanghai Yuanye Biotechnology Co., Ltd (Shanghai, China). *β*-Nicotinamide adenine dinucleotide phosphate (NADP) and lucifer yellow were provided by Sigma (St. Louis, MO, USA). Rat liver microsomes were purchased from BD (Franklin Lakes, NJ, USA). Dulbecco’s modified Eagle’s medium (DMEM) and non-essential amino acid (NEAA) solution were purchased from Thermo Scientific Corp. (Logan, UT, USA). Fetal bovine serum (FBS) was obtained from GIBCO BRL (Grand Island, NY, US). Penicillin G (10,000 U/mL) and streptomycin (10 mg/mL) were purchased from Amresco (Solon, OH, USA). Hanks’ balanced salt solution (HBSS) was purchased from GIBCO (Grand Island, NY, USA). Acetonitrile and methanol were purchased from Fisher Scientific (Fair Lawn, NJ, USA). Ultrapure water was prepared with a Milli-Q water purification system (Millipore, Billerica, MA, USA). All other chemicals were of analytical grade or better.

### Animal experiments

Male Sprague-Dawley rats weighing 230–250 g were provided by the experimental animal center of the Shanghai University of TCM (Shanghai, China). Rats were bred in a breeding room at 25 °C with 60 ± 5% humidity and a 12 h dark–light cycle. Tap water and normal chow were given *ad libitum*. All of the experimental animals were housed under the above conditions for a three-day acclimation period and fasted overnight before the experiments. All experimental procedures and protocols were reviewed and approved by the Animal Care and Use Committee of Shanghai University of TCM and were in accordance with the National Institutes of Health guidelines regarding the principles of animal care.

### *In vivo* pharmacokinetic study

To evaluate the effects of psoralen on the pharmacokinetics of anastrozole, the rats were divided into two groups of six animals each. The test group was pretreated with psoralen by oral gavage at a dose of 20 mg/kg/day (dissolved directly in normal saline containing 0.5% methylcellulose at a concentration of 20 mg/mL) for 10 days before the administration of anastrozole. Next, anastrozole (dissolved in normal saline solution at a concentration of 0.02 mg/mL) were orally administered to rats by gavage at a dose of 0.5 mg/kg. Blood samples (200 μL) were collected into heparinized tubes via the *oculi chorioideae* vein at 0.083, 0.33, 0.5, 1, 2, 4, 6, 8, 10, 12, 24 and 36 h after the oral administration of anastrozole. The blood samples were centrifuged at 3500 rpm for 5 min. The plasma samples that were obtained were stored at −40 °C until analysis.

### Instruments and conditions

The analysis was performed on an Agilent 1290 series liquid chromatography system and an Agilent 6460 triple-quadrupole mass spectrometer (Palo Alto, CA, USA). The chromatographic analysis of anastrozole was performed on a Waters Xselect HSS PFP column (3.0 × 100 mm, i.d.; 3.5 μm, USA) at room temperature (25 °C). The mobile phase was water (containing 0.1% formic acid) and methanol (30:70, v:v) with isocratic elution at a flow rate of 0.3 mL/min and the analysis time was 4 min. The injection volume was 2 μL.

The mass scan mode was the positive MRM mode. The precursor ion and product ion were *m/z* 294.2 → 225.1 for anastrozole and *m/z* 345.8 → 197.8 for omeprazole, respectively. The collision energy for anastrozole and internal standard were 20 and 15 eV, respectively. The MS/MS conditions were optimized as follows: fragmentor, 110 V; capillary voltage, 4 kV; nozzle voltage, 500 V; nebulizer gas pressure (N_2_), 40 psig; drying gas flow (N_2_), 10 L/min; gas temperature, 350 °C; sheath gas temperature, 400 °C and sheath gas flow, 11 L/min.

### Preparation of calibration standards, quality control and internal standard

The stock solution of anastrozole was prepared in methanol at a concentration of 10 mg/mL and the stock solution of omeprazole was prepared in methanol at a concentration of 1 mg/mL. The internal standard stock solution was diluted to 10 ng/mL before use. A series of standard working solutions were obtained by further diluting the stock solution of anastrozole with methanol. The calibration standard samples for anastrozole (1, 2, 5, 10, 20, 50 and 100 ng/mL) were prepared by spiking 20 μL of the working standard solution into 100 μL blank rat plasma. A 180 μL aliquot of internal standard methanol solution was added and vortexed for 60 s to mix in a 1.5 mL polypropylene tube, and were centrifuged at 12,000 rpm for 10 min. The quality control (QC) samples for anastrozole were prepared at low (2 ng/mL), medium (20 ng/mL) and high (75 ng/mL) concentrations in the same way as the plasma samples for calibration. The QC samples were stored at −4 °C until analysis.

### Preparation of rat plasma samples

To 100 μL aliquot of a plasma sample, 20 μL methanol and 180 μL internal standard methanol solution (10 ng/mL) were added and vortexed for 60 s to mix in a 1.5 mL polypropylene tube, and were centrifuged at 12,000 rpm for 10 min. The supernatant was removed into an injection vial, and a 2 μL aliquot was injected into the LC-MS/MS system for analysis.

### Method validation

#### Specificity

Specificity was investigated by analyzing six individual blank rat plasma samples which were compared to those obtained by the spiking analyte and IS into the corresponding blank plasma sample to monitor interference.

#### Linearity and sensitivity

For the calibration curve, nine concentrations of calibration standards (1, 2, 5, 10, 20, 50 and 100 ng/mL) were processed and determined as described above. The calibration curves for anastrozole were constructed by plotting peak area ratios of the analyte to IS against plasma concentrations. The lower limit of quantification (LLOQ) was determined as the concentration of the analyte with a signal-to-noise ratio of 10.

#### Precision and accuracy

To determine intra-day precision and accuracy, six replicates of QC samples at low, medium and high concentration levels (2, 20 and 75 ng/mL) were prepared and analyzed on the same day. Inter-day precision and accuracy were evaluated on three independent days. The intra- and inter-day precisions were expressed as the RSD value and the accuracy as the RE value.

#### Extraction recovery and matrix effect

The extraction recovery was determined by calculating the ratio of QC samples obtained against those originally spiked in the blank plasma; this step was replicated six times. The matrix effect was evaluated by comparing the solution spiked with the blank processed matrix with the solution at three different QC concentrations; this step was replicated six times. The extraction recovery and matrix effect of the IS were also determined.

#### Stability

The short-term stability was evaluated by determining QC samples at room temperature for 6 h. The auto-sampler stability was detected in the auto-sampler after preparation for 12 h. The long-term stability was assessed by storing the QC samples at −20 °C for 30 days. The freeze–thaw stability was determined through three freeze–thaw cycles on consecutive days.

### Data analysis

The pharmacokinetic parameters, including the area under the plasma concentration-time curve (*AUC*), maximal plasma concentration (*C*_max_), the time for the maximal plasma concentration (*T*_max_) and the mean residence time (*MRT*) were calculated using the DAS 3.0 pharmacokinetic software (Chinese Pharmacological Association, Anhui, China).

The differences between the mean values were analyzed for significance using a one-way analysis of variance (ANOVA). Values of *p <* 0.05 were considered to be statistically significant.

### Cell culture

The Caco-2 cell line was obtained from the American Type Culture Collection (Manassas, VA, USA). The Caco-2 cells were cultured in DMEM high glucose medium containing 15% FBS, 1% NEAA and 100 U/mL penicillin and streptomycin. The cells were cultured at 37 °C with 5% CO_2_. For transport studies, the cells at passage 40 were seeded on transwell polycarbonate insert filters (1.12 cm^2^ surface, 0.4 μm pore size, 12 mm diameter; Corning Costar Corporation, MA, USA) in 12-well plates at a density of 1 × 10^5^ cells/cm^2^. Cells were allowed to grow for 21 days. For the first seven days, the medium was replaced every two days, and daily thereafter. The transepithelial electrical resistance (TEER) of the monolayer cells was measured using Millicell ERS-2 (Millipore Corporation, MA, USA), and TEER exceeding 400 Ω cm^2^ was used for the flux experiment. The integrity of the Caco-2 monolayers was confirmed by the paracellular flux of Lucifer yellow, which was less than 1% per hour. The alkaline phosphatase activity was validated using an Alkaline Phosphatase Assay Kit. The qualified monolayers were used for transport studies.

### Effects of psoralen on the transport of anastrozole in Caco-2 cell transwell model

Before the transport experiments, the cell monolayers were rinsed twice using warm (37 °C) Hanks’ balanced salt solution (HBSS), and the Caco-2 cell transwell model were incubated at 37 °C for 20 min. After incubation, the Caco-2 cell transwell model were incubated with anastrozole in fresh incubation medium added on either the apical or basolateral side for the indicated times at 37 °C. The volume of incubation medium on the apical and basolateral sides was 0.5 mL and 1.5 mL, respectively. A 100 μL aliquot of the incubation solution was withdrawn at the indicated time points from the receiver compartment and replaced with the same volume of fresh pre-warmed HBSS buffer. The efflux activity of *P-gp* was validated using a typical *P-gp* substrate digoxin (25 μM). The effects of psoralen or verapamil (*P-gp* inhibitor) on the transport of anastrozole were investigated by adding 50 μM psoralen or verapamil to both sides of the cell monolayers and pre-incubating the sample at 37 °C for 2 h. In addition, the effects of psoralen on the efflux of digoxin (25 μM) were also investigated. The permeability of anastrozole (1 μM) (which was validated for no toxicity for Caco-2 cells within 2 h) in all of the above conditions for both directions, i.e., from the apical (AP) side to the basolateral (BL) side and from the BL side to the AP side, was measured after incubation for 30, 60, 90 and 120 min at 37 °C.

The apparent permeability coefficient (*P*_app_) was calculated using the equation of Artursson and Karlsson (1991):

$$P_{app}=\left(\Delta Q/\Delta t\right)*\left[1/\left(A*C_{0}\right)\right]$$

where *P*_app_ is the apparent permeability coefficient (cm/s), ΔQ/Δt (μmol/s) is the rate at which the compound appears in the receiver chamber, *C*_0_ (μmol/L) is the initial concentration of the compound in the donor chamber and A (cm^2^) represents the surface area of the cell monolayer. Data were collected from three separate experiments, and each was performed in triplicate.

### Effects of psoralen on the metabolic stability of anastrozole in rat liver microsomes

Rat liver microsomes were used to determine the phase I metabolic stability of anastrozole. The assay conditions and reaction mixtures were similar to those reported previously (Dong et al. 2015; Gong et al. 2015). In brief, except for the NADPH-generating system, 30 μL of rat liver microsomes (20 mg/mL), 12 μL of anastrozole solution (100 μM) and 1113 μL of PBS buffer (0.1 M, pH 7.4) were added to the centrifugation tubes on ice. There was a 5 min pre-incubation step at 37 °C before initiating the reaction by adding the NADPH-generating system (45 μL) to the microsomal suspension. The effects of psoralen on the metabolic stability of anastrozole were investigated by adding 2 mM of psoralen (12 μL) to rat liver microsomes and pre-incubating them for 30 min at 37 °C, followed by the addition of the NADPH-generating system. Aliquots of 100 μL were collected from the reaction mixtures at 0, 1, 3, 5, 15, 30 and 60 min after the addition of anastrozole, and 200 μL of ice-cold acetonitrile containing syringin was added to terminate the reaction. The subsequent sample preparation method was the same as the plasma sample preparation method, and the concentration of anastrozole was determined by LC-MS/MS.

The *in vitro* half-life (*t*_1/2_) was obtained using the equation: *t*_1/2 _*=* 0.693/*k*; V (μL/mg)=volume of incubation (μL)/protein in the incubation (mg); Intrinsic clearance (Clint) (μL/min/mg protein)=v × 0.693/*t*_1/2_.

## Results

### Method validation

**Figure 1.**
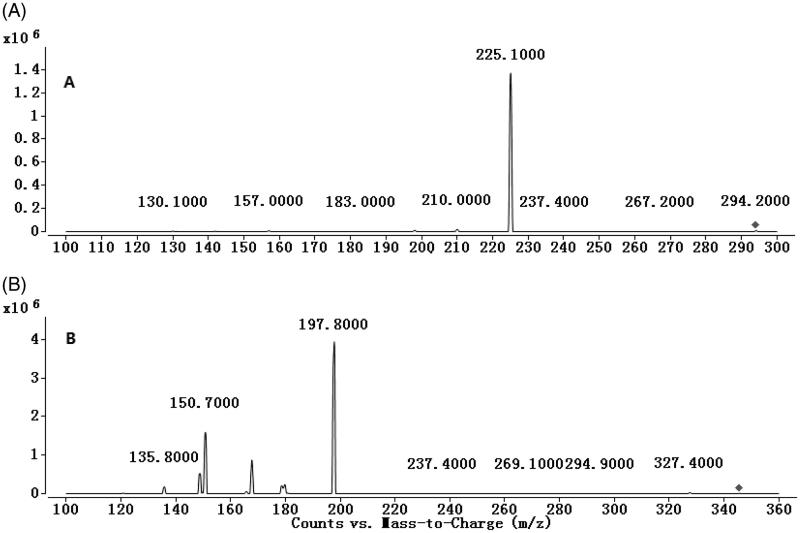
Mass spectra of anastrozole (A) and omeprazole (B).

**Figure 2.**
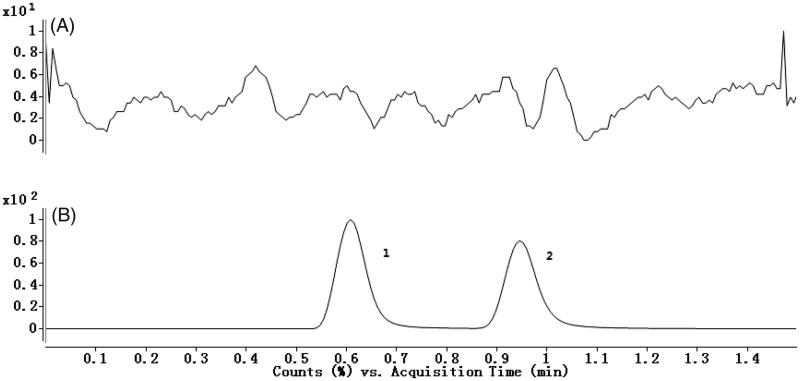
(A) Representative chromatograms of blank plasma; (B) Blank plasma spiked with anastrozole and omeprazole; 1: anastrozole, 2: omeprazole.

To develop a sensitive and accurate LC-MS/MS method for the determination of anastrozole in rat plasma, quantitative analysis was performed using the MRM mode owing to its high selectivity and sensitivity. The precursor and product ions were *m/z* 294.2 → 225.1 for anastrozole and *m/z* 345.8 → 197.8 for omeprazole. The mass ion spectra of anastrozole and omeprazole are shown in Figure 1. The MS/MS conditions were optimized to achieve better sensitivity and selectivity. To obtain the appropriate retention time and response, methanol, acetonitrile, water and formic acid were tested as mobile phases. After optimization, 0.1% formic acid was found to enhance the efficiency of ionization and obtain a better intensity than pure water for all compounds tested. Blank plasma, plasma spiked with anastrozole and omeprazole are shown in Figure 2. No significant interference substances were observed at the retention time of anastrozole and omeprazole in the plasma samples.

The calibration curve for anastrozole was constructed by plotting peak area ratios of the analyte to IS against plasma concentrations using a linear least-squares regression model. Linearity for determining anastrozole in spiked rat plasma was prepared by nine calibration standards in five independent runs. The calibration curves were obtained with correlation coefficients (*r*) more than 0.999 between 1 and 100 ng/mL of anastrozole. The LLOQ was set at 1 ng/mL for anastrozole in rat plasma samples.

The intra- and inter-day precision of the method was assessed at three concentration levels of spiked analyte in triplicate and was verified by determining the ratios of the peak areas of these compounds to the internal standard with relative standard deviation (RSD) as listed in Table 1. The precision of this method was no more than 10% relative standard deviations (RSD) for anastrozole. The accuracy ranged from −8.37 to 9.00% for anastrozole, demonstrating the satisfactory precision and accuracy of the instrumentation.

**Table 1.** The precision and accuracy of anastrozole in plasma samples (*n* = 6).

Analyte	Nominal concentration (ng/mL)	Intra-day			Inter-day		
		concentration measured (ng/mL)	Precision (%, RSD)	Accuracy (%, RE)	concentration measured (ng/mL)	Precision (%, RSD)	Accuracy (%, RE)
Anastrozole	2	1.87	7.86	−6.50	2.18	8.16	9.00
	20	21.87	6.37	9.35	18.69	6.04	−6.55
	75	68.72	5.59	−8.37	81.67	7.12	8.89

To achieve high recovery efficiency in sample preparation, the direct precipitation method was used for its convenience and low matrix effect. Next, the extraction recovery of the precipitation solvents (methanol and acetonitrile) was investigated. As shown in Table 2, the extraction efficiency of anastrozole and IS exceeded 90% using methanol as extraction solution, suggesting that it was an ideal precipitation agent.

**Table 2.** Extraction recovery and matrix effect of anastrozole in plasma samples (*n* = 6).

Analyte	Nominal concentration (ng/mL)	Extraction recovery (%)	RSD (%)	Matrix effect (%)	RSD (%)
Anastrozole	2	90.67	7.28	90.01	6.37
	20	91.05	5.59	87.36	8.15
	75	92.51	6.12	89.57	7.38

As shown in Table 2, the matrix effect of anastrozole was between 87.36 and 90.01%. These results indicate that the method was reliable and no significant matrix effect was observed.

Analyte stability was assessed under various conditions. The results indicated that anastrozole under these conditions were all stable in plasma samples (RE <15%), which are shown in Table 3.

**Table 3.** Stability of anastrozole in plasma samples (*n* = 3).

Analyte	Nominal concentration (ng/mL)	Stability (%, RE)			
		Short-term (6 h at room temperature)	Auto-sampler (12 h)	Long-term (30 days at −20 °C)	3 freeze–thaw cycles at −20 °C
Anastrozole	2	7.35	7.31	5.56	9.22
	20	5.58	−5.28	8.10	6.85
	75	−9.05	7.55	7.09	−7.26

Method validation was also conducted in Caco-2 cell transport media and rat liver microsome samples, and the results indicated that this method could also be used for the analysis of celastrol in Caco-2 cell transport media and rat liver microsomes samples.

### Effects of psoralen on the pharmacokinetics of anastrozole

**Figure 3.**
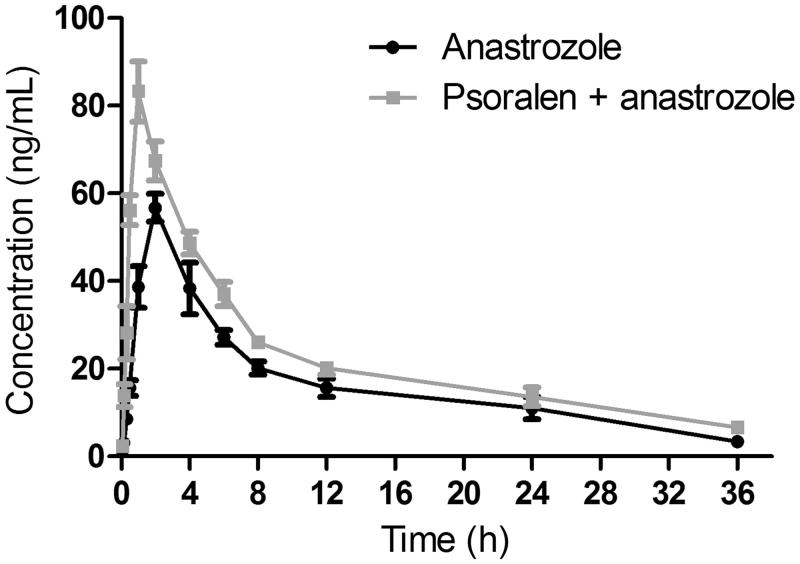
Pharmacokinetic profiles of anastrozole in male Sprague-Dawley rats after oral administration of 0.5 mg/kg anastrozole with or without psoralen (20 mg/kg/day for 10 days) pretreatment. Each point with a bar represents the mean ± S.D. of six rats.

The mean plasma concentration-time curves of anastrozole after oral administration of anastrozole or oral administration of anastrozole with the pre-treatment of psoralen are presented in Figure 3. The pharmacokinetic parameters are shown in Table 4.

**Table 4.** Pharmacokinetic parameter of puerarin in rats after oral administration of anastrozole (0.1 mg/kg; *n* = 6, Mean ± S.D.) with or without psoralen pretreatment.

Parameter	Control	Psoralen pretreatment
*T*_max_ (h)	1.95 ± 0.12	1.03 ± 0.08*
*C*_max_ (ng/mL)	56.74 ± 3.17	83.26 ± 6.87*
*t*_1/2_ (h)	10.80 ± 1.05	14.29 ± 1.38*
*AUC*_(0-inf)_ (μg h/L)	590.38 ± 76.91	803.26 ± 92.27*

*p < 0.05 indicate significant differences from the control.

As shown in Table 4, when the rats were pretreated with psoralen (20 mg/kg) for 10 days, the *C*_max_ of anastrozole increased from 56.74 ± 3.17 to 83.26 ± 6.87 ng/mL, the difference was significant (*p* < 0.05), and the *AUC*_(0-inf)_ also decreased significantly (*p* < 0.05). The *T*_max_ decreased from 1.95 ± 0.12 to 1.03 ± 0.08 h. The *t*_1/2_ value of anastrozole increased from 10.80 ± 1.05 to 14.29 ± 1.38 h, the difference was also significant (*p* < 0.05). The oral clearance of anastrozole decreased significantly from 0.16 ± 0.02 to 0.11 ± 0.01 L/h/kg (*p* < 0.05). These results indicate that psoralen could increase the system exposure of anastrozole in rats when psoralen and anastrozole are co-administered.

### Effects of psoralen on the bidirectional transport of anastrozole in Caco-2 cell transwell model

**Figure 4.**
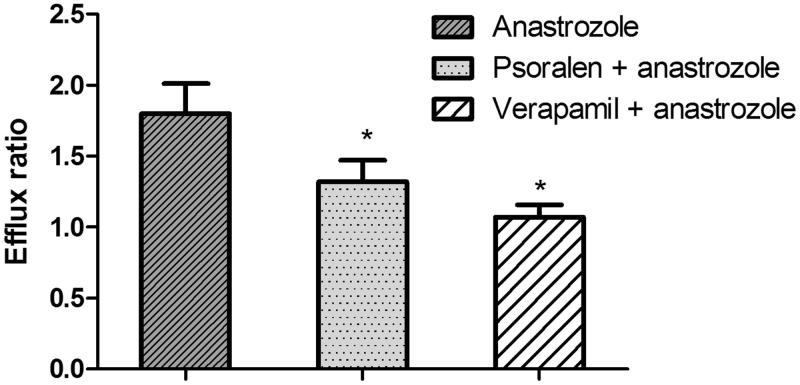
Effects of psoralen or verapamil on the efflux ratio of anastrozole in the Caco-2 cell monolayer model. Each bar represents the average ± S.D. of three determinations. *p < 0.05 indicate significant differences from the anastrozole group.

To investigate the effects of psoralen on the transport of anastrozole, the Caco-2 cell transwell model was utilized. To validate the efflux activity of *P-gp*, a typical *P-gp* substrate digoxin was used, and the results indicated that the efflux ratio of digoxin was 10.58 (*P*_appAB_: 1.18 ± 0.13 × 10^−7 ^cm/s; *P*_appBA_: 1.25 ± 0.10 × 10^−6 ^cm/s), which was abrogated in the presence of a typical *P-gp* inhibitor verapamil. After pre-treatment with psoralen for 24 h, the efflux ratio of digoxin decreased from 10.58 to 3.58 (*P*_appAB_: 1.18 ± 0.13 × 10^−7 ^cm/s; *P*_appBA_: 4.22 ± 0.10 × 10^−7 ^cm/s). The results indicated that the efflux activity of *P-gp* was qualified for the experiment. Next, the transport of 1 μM of anastrozole across the Caco-2 cell transwell model was investigated in this study. As shown in Figure 4, The *P*_appAB_ and *P*_appBA_ were 1.05 ± 0.11 × 10^−6 ^cm/s and 1.89 ± 0.17 × 10^−6 ^cm/s, respectively. The *P*_appBA_ was much higher than the *P*_appAB_, and the efflux ratio was 1.80, which indicated that efflux transporters might be involved in the transport of anastrozole. After that step, the transport studies were performed in the presence of psoralen or verapamil to determine its effects on the transport of anastrozole. In the presence of 50 μM of psoralen, the *P*_appAB_ decreased (1.28 ± 0.15 × 10^−6 ^cm/s), whereas *P*_appBA_ increased (1.69 ± 0.23 × 10^−6 ^cm/s). The efflux ratio increased from 1.88 to 1.32 (*p* < 0.05). However, in the presence of verapamil (50 μM), a typical *P-gp* inhibitor, the efflux ratio decreased from 1.80 to 1.07 (*P*_appAB_: 1.35 ± 0.14 × 10^−6 ^cm/s; *P*_appBA_: 1.45 ± 0.16 × 10^−6 ^cm/s) (*p* < 0.05). These results indicated that *P-gp* was involved in the transport of anastrozole in the Caco-2 cell transwell model, and psoralen could increase the absorption of anastrozole and decrease its efflux of anastrozole by inhibiting the activity of *P-gp*.

### Effects of psoralen on the metabolic stability of anastrozole in rat liver microsome incubation systems

The effects of psoralen on the metabolic stability of anastrozole were investigated using rat liver microsomes incubation systems. The metabolic half-life of anastrozole is 22.06 ± 2.58 min, and the intrinsic clearance rate of anastrozole is 62.83 ± 7.16 μL/min/mg protein. In the presence of psoralen, the metabolic half-life of anastrozole (31.52 ± 3.07 min) was prolonged, and the intrinsic clearance rates (43.97 ± 9.31 μL/min/mg protein) were decreased. These results indicate that psoralen significantly (*p* < 0.05) decreases the intrinsic clearance rates of anastrozole in rat liver microsomes.

## Discussion

The pharmacokinetic experiments showed that psoralen could increase the system exposure of anastrozole in rats. To investigate its potential mechanism, the Caco-2 cell transwell experiments were used and the results indicated that *P-gp* was involved in the transport of anastrozole in the Caco-2 cell transwell model, and psoralen could increase anastrozole intestine absorption through inhibiting the activity of *P-gp*.

Second, the metabolism clearance was also investigated using rat liver microsome incubation systems and the results revealed that psoralen could decrease its metabolism clearance in rat liver through inhibiting the activity of CYP3A4.

Anastrozole is metabolized predominantly via CYP3A and is also a substrate of *P-gp*, an efflux transporter that can limit the oral absorption of anastrozole. Consequently, co-administration of foods or drugs with influence on CYP3A and/or *P-gp* may affect the pharmacokinetics of anastrozole.

Therefore, results of this study indicate that when the rats were pre-treated with psoralen, the system exposure of anastrozole would be increased significantly. The results indicate that the herb-drug interaction between psoralen and anastrozole might occur when they were co-administered.

These changes could enhance anastrozole efficacy but could also make drug accumulation to increase the occurrence of adverse effects, so it was suggested that the dosage should be adjusted or the drug concentration in plasma should be monitored if psoralen and anastrozole are co-administered in the clinics.
